# *Lin28B* overexpression decreases *let-7b* and *let-7g* levels and increases proliferation and estrogen secretion in Dolang sheep ovarian granulosa cells

**DOI:** 10.5194/aab-66-217-2023

**Published:** 2023-07-28

**Authors:** Zhiyuan Sui, Yongjie Zhang, Zhishuai Zhang, Chenguang Wang, Xiaojun Li, Feng Xing

**Affiliations:** 1 Key Laboratory of Tarim, Animal Husbandry Science and Technology, Xinjiang Production & Construction Corps, Alar, Xinjiang 843300, China; 2 College of Animal Science and Technology, Tarim University, Alar, Xinjiang 843300, China

## Abstract

Although ovine puberty initiation has been previously studied, the mechanism by which the RNA-binding protein Lin28B affects this process has not been investigated. The present study aimed to investigate the effects of *Lin28B* overexpression on *let-7b*, *let-7g*, cell proliferation, and estrogen secretion in Dolang sheep ovine ovarian granulosa cells. In this study, a *Lin28B* vector was constructed and transfected into ovarian granulosa cells using liposomes. After 24, 48, and 72 h of overexpression, quantitative real-time PCR (qRT-PCR) was used for measuring *let-7b* and *let-7g* microRNA (miRNA) levels, and estrogen secretion was measured using the enzyme-linked immunosorbent assay (ELISA). A CCK-8 (Cell Counting Kit-8) kit was used for evaluating cell viability and proliferation in response to *Lin28B* overexpression at 24 h. The results showed that the expression of *let-7b* and *let-7g* decreased significantly after *Lin28B* overexpression, and the difference was consistent over different periods. The result of ELISA showed that estradiol (E2) levels significantly increased following *Lin28B* overexpression. Additionally, *Lin28B* overexpression significantly increased the cell viability and proliferation. Therefore, the *Lin28B*–let-7 family axis may play a key role in the initiation of female ovine puberty.

## 1 Introduction

Puberty is the first estrus period in female animals during which ovulation occurs. Puberty is related to the hypothalamus–pituitary–gonadal axis regulation and the effect of environmental and genetic factors on the coordinating functions of luteinizing and follicle-stimulating hormones (Meeran et al., 2003; Redmond et al., 2011; Wankowska et al., 2008; Pool et al., 2020; Rosa and Bryant, 2003). Puberty is affected by various factors, including genetic mechanisms, nutrient levels, and light duration, all of which affect the timing of puberty (Greives et al., 2007; Suttie et al., 1985). Studies have shown that the *Lin28B* gene expression in the hypothalamus plays an important role in puberty initiation in mammals (Tommiska et al., 2010).

Lin28B is a highly conserved RNA-binding protein first discovered in *Caenorhabditis elegans*, and this heterochronic gene regulates nematode development from the larval to adult stages (Ambros, 1989). *Lin28B* was first cloned in human hepatocellular carcinoma. It is located on chromosome 6. Lin28B has a very long 3 ${}^{\prime }$ -UTR (untranslated region) and a complementary site for let-7 microRNAs (Guo et al., 2006).

MicroRNAs (miRNAs), a type of endogenous non-coding small RNA composed of 20–30 nucleotides, are involved in cell development, proliferation, differentiation, and apoptosis by targeting specific mRNAs, mediating translational repression, or degrading mRNAs (Baek et al., 2008; Bartel, 2004; Hwang and Mendell, 2006). *Lin28B* inhibits the biogenesis of various miRNAs, including the let-7 family miRNAs. Let-7 miRNAs regulate genes related to cell growth and differentiation (Peng et al., 2011). Among them, let-7b inhibits cyclin D1 expression, which regulates the self-renewal of embryonic stem cells and the proliferation and tumorigenicity of cancer cells (Schultz et al., 2008; Xu et al., 2009; F. Yu et al., 2007). *Lin28B* suppresses miRNA *let-7b* expression to promote CD44 ${}^{+}$ /*Lin28B* ${}^{+}$ human pancreatic cancer stem cell proliferation and invasion (Shao et al., 2015).

In mammals, granulosa cell (GC) proliferation plays an important regulatory role in determining follicle fate and maturation (Douville and Sirard, 2014; Khan et al., 2016; Saatcioglu et al., 2016). GCs produce estradiol (E2), which supports their survival and proliferation and promotes follicle maturation (Chou and Chen, 2018). Mammalian follicle development is a key process within the ovary, to which GCs directly contribute through their proliferation and growth (Lv et al., 2019). In this context, previous reports indicate interactions among E2, *Lin28B*, and the let-7 family; 17- β -estradiol and *let-7a*–*Lin28B* axes synergistically affect the occurrence and development of adenomyosis (Huang et al., 2021). Additionally, treating MCF-7 cells with 17- β -estradiol (E2) resulted in rapid and specifically reduced *let-7g* expression (Qian et al., 2011).

Thus far, relatively few studies have reported on *Lin28B* and ovine puberty. In the present study, an overexpression vector was constructed from the Dolang sheep *Lin28B* sequence and transfected into sheep ovarian granulosa cells. This sequence was overexpressed for identifying its effects on *let-7b*, *let-7g*, cell proliferation, and estrogen secretion. Thus, this study lays a foundation for a better understanding of how *Lin28B* may contribute to the initiation of puberty in sheep.

## 2 Materials and methods

### 2.1 Cell collection and culture

Ovaries of Dolang sheep (approximately 3.5 months old) were collected immediately after slaughter and placed in normal saline at 37 ${}^{\circ }$ C and brought back to the laboratory for processing. The cumulus–oocyte complex (COC) was aspirated from follicles with a diameter of 3–8 mm. GCs were collected after serial pipetting, and the follicular fluid and medium were mixed and injected into a sterile 15 mL centrifuge tube. The supernatant was discarded after centrifugation at 175 g for 5 min. Subsequently, 3 mL of DMEM (Dulbecco's Modified Eagle Medium) was added, stirred gently to mix, and centrifuged at 175 g for 5 min, and the supernatant was discarded. A freshly prepared complete medium (89 % DMEM + 10 % fetal bovine serum + 1 % penicillin / streptomycin) (Gibco, USA) was added, mixed, and transferred to Petri dishes. Isolated ovarian granulosa cells were cultured at 37 ${}^{\circ }$ C under 5 % CO ${}_{2}$ for subsequent experiments.

### 2.2 Immunofluorescence assay

Cell slides were placed into 24-well cell culture plates (2 × 10 ${}^{4}$ cells per well), and 1 mL of culture medium was added. The cells were incubated for 24 h in an incubator. After the cells had attached to the slides, the medium was aspirated, and the cells were washed once with PBS (phosphate-buffered saline) (Gibco, USA), fixed with 4 % paraformaldehyde (Solarbio, Beijing, China) for 30 min at 4 ${}^{\circ }$ C, washed three times with PBS for 5 min each, and blocked with a blocking solution (0.5 % Triton X-100 mixed with PBS 1 : 1, plus 10 % goat serum) (Solarbio, Beijing, China) at room temperature for 2 h. The cell slides were incubated with a primary antibody (FSHR antibody : PBS = 1 : 100) (22665-1-AP, Proteintech, Wuhan, China) at 4 ${}^{\circ }$ C for 24 h. Subsequently, they were incubated with Goat anti-Rabbit IgG (H + L) Cross-Adsorbed (IgG : PBS = 1 : 500) (SA00006-3, Proteintech, Wuhan, China) for 2 h at room temperature in the dark and washed three times with PBS for 5 min each. The cells were stained with DAPI (DAPI : PBS = 1 : 1000) (Solarbio, Beijing, China) for 5 min and washed three times with PBS for 5 min each. One drop of Fluoromount-G (SouthernBiotech, USA) was dropped on the slide, and the side with cells was covered. Images were acquired using a fluorescence microscope (DS-Ri2; Nikon, Japan) and examined with a 100× objective.

### 2.3 Construction of *Lin28B* overexpression vector

The pEGFP-N1 vector was digested with NheI and EcoRI. The 754 bp coding sequence of *Lin28B* was amplified from the cDNA of Dolang sheep using qRT-PCR (Takara, Dalian, China) (Xing et al., 2019a). The amplified cDNA was digested with NheI and EcoRI and ligated into the pEGFP-N1 vector to generate pEGFP-N1–*Lin28B*, which was verified by DNA sequencing.

**Figure 1:**
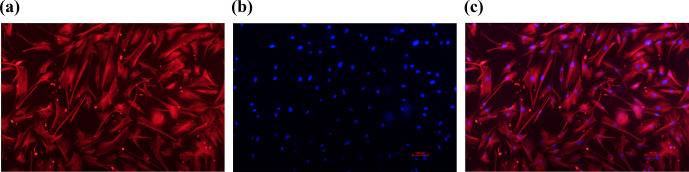
Identification of sheep granulosa cells (GCs). **(a)** The red marker indicates cells expressing FSHR. **(b)** The blue marker indicates DAPI-stained nuclei. **(c)** Merge is a red fluorescently labeled FSHR with a blue fluorescently DAPI overlay. Bar: 100 µ m.

**Figure 2:**
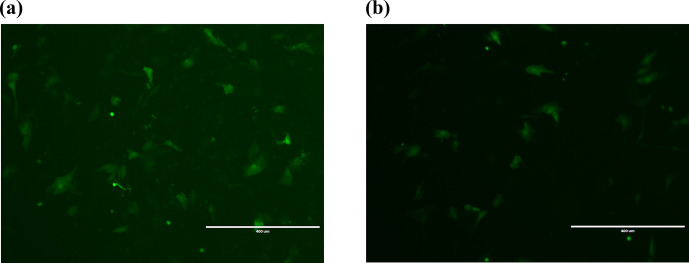
Transfection 24 h cell fluorescence map. **(a)** Fluorescence detection graph of empty-vector group 24 h after transfection. **(b)** Fluorescence detection graph of overexpression of *Lin28B* groups 24 h after transfection. Bar: 400 µ m.

### 2.4 Cell transfection

Cultured Dolang sheep ovary granulosa cells were suspended with PBS for later use. Trypan blue staining solution (0.4 %) was added to the cell suspension at a cell dye ratio of 1 : 1 ( v/v ), and the cells were counted using a hemocytometer. According to the manufacturer's instructions for Lipofectamine 3000, $1\times 10^{6}$ ovarian granulosa cells were plated in six-well plates and cultured for 24 h. The cells were transfected using a plasmid : transfection reagent ratio of 1 : 3. Cell transfection was divided into three periods: 24 h (0–24 h), 48 h (0–48 h), and 72 h (0–72 h). The cells were grouped as target gene, empty vector, and untreated cells. These experiments were run in triplicate. The medium from each time period was collected for measuring estrogen secretion, and cell RNA was extracted by TRIzol–chloroform extraction for quantitative real-time PCR (qRT-PCR).

### 2.5 Western blotting

The total cell protein was extracted using a kit (TransGen Biotech, China), and the protein concentration was detected using a BCA kit (TransGen Biotech). A 10 % separating gel and 5 % stacking gel were prepared for electrophoresis. The band was excised and transferred to the membrane, following which it was incubated overnight at 4 ${}^{\circ }$ C with the primary antibodies, namely anti-Lin28B (1 : 1000, Abcam, UK) and anti-ACTB (1 : 5000, Proteintech). The membrane was then incubated with an enzyme-conjugated secondary antibody for 2 h at 37 ${}^{\circ }$ C. The bands were detected using an ELC luminescence kit (Beyotime, Jiangsu, China).

### 2.6 Reverse transcription and expression of miRNA

The expression of *Lin28B*, *let-7b*, and *let-7g* in ovarian granulosa cells 24, 48, and 72 h after transfection was assessed using qRT-PCR.

For *Lin28B* mRNA expression, ACTB was selected as the reference gene for normalizing mRNA levels. The primer sequences for *Lin28B* and ACTB mRNA are presented in Table 1.

**Table 1:** Primers used for qRT-PCR analysis.

Designation	Forward (5 ${}^{\prime }$ –3 ${}^{\prime }$ )	Reverse (5 ${}^{\prime }$ –3 ${}^{\prime }$ )
*Lin28B*	ACCAAAGGGAGACAGATGCTACA	CACCATGTGCGTGATGCTCT
*let-7b*	ACCTGAGGTAGTAGGTTGTGTGGT	miRNA qRT-PCR TB Green Kit
*let-7g*	GGCACCTGAGGTAGTAGTTTGTACAGT	miRNA qRT-PCR TB Green Kit
ACTB	GCAGATGTGGATCAGCAAGC	TCTCGTTTTCTGCGCAAGTT
U6	GGAACGATACAGAGAAGATTAGC	TGGAACGCTTCACGAATTTGCG

For *let-7b* and *let-7g* quantification, cDNA was synthesized using a miRNA RT Kit (Takara, Dalian, China). The mature sequences of *let-7b* and *let-7g* were obtained using miRBase (https://www.mirbase.org/, last access: 18 April 2022) and were used to design the primers. The forward primers for *let-7b* and *let-7g* were designed, whereas the reverse primers were included in the Takara miRNA RT Kit. U6 was selected as a reference gene. Table 1 presents the primer sequences used for *let-7b*, *let-7g*, and U6 miRNA.

The qRT-PCR was performed using the Mir-X miRNA qRT-PCR TB Green Kit (Takara, Dalian, China). All experiments were run on an Eppendorf device (Eppendorf, Germany). Each sample and assay were run in triplicate. The levels of *Lin28B*, *let-7b*, and *let-7g* were calculated using the 2 ${}^{-\Delta \Delta \mathrm{CT}}$ method.

### 2.7 Cell proliferation

Cell proliferation was measured using a CCK-8 (Cell Counting Kit-8) assay (Beyotime, Jiangsu, China). Cells ( $5\times 10^{3}$ ) were resuspended in complete DMEM and seeded into a 96-well culture plate. The plate was incubated at 37 ${}^{\circ }$ C for 24 h. The incubator was maintained in a humidified atmosphere of 5 % CO ${}_{2}$ . WST-8 cell proliferation reagent was added, and the plate was incubated for an additional 2 h. Absorbance at 450 nm was measured using a microplate reader (BioTek, USA) to analyze the effect of *Lin28B* overexpression on cell proliferation.

### 2.8 Hormone determination

ELISA (Jining, Shanghai, China) was used for detecting E2 concentration in the cell culture media collected at 24, 48, and 72 h.

### 2.9 Statistical analysis

SPSS 26.0 software (IBM, Chicago, IL, USA) was used for statistical analysis. All experiments were repeated at least three times, and the experimental results are presented as the mean ± standard error. A t test was used for comparing two groups, and a one-way ANOVA was used for testing the differences among multiple groups. Statistical significance was set at P<0.05 .

**Figure 3:**
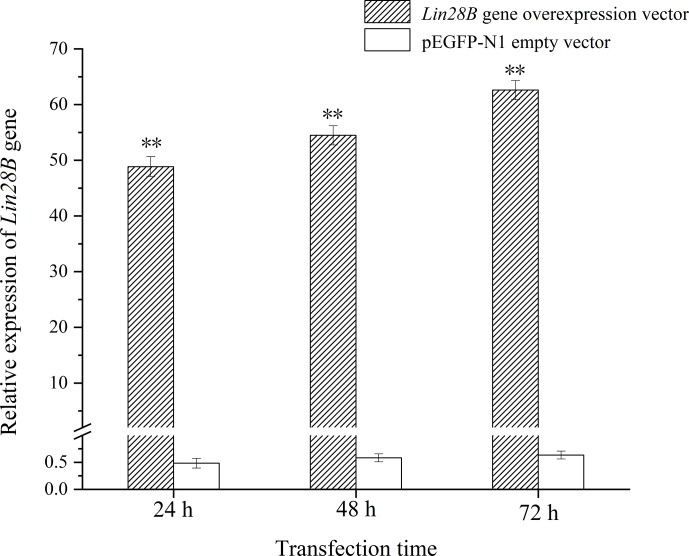
Changes in *Lin28B* mRNA expression at 24, 48, and 72 h after transfection. ${}^{**}$ P < 0.01.

**Figure 4:**
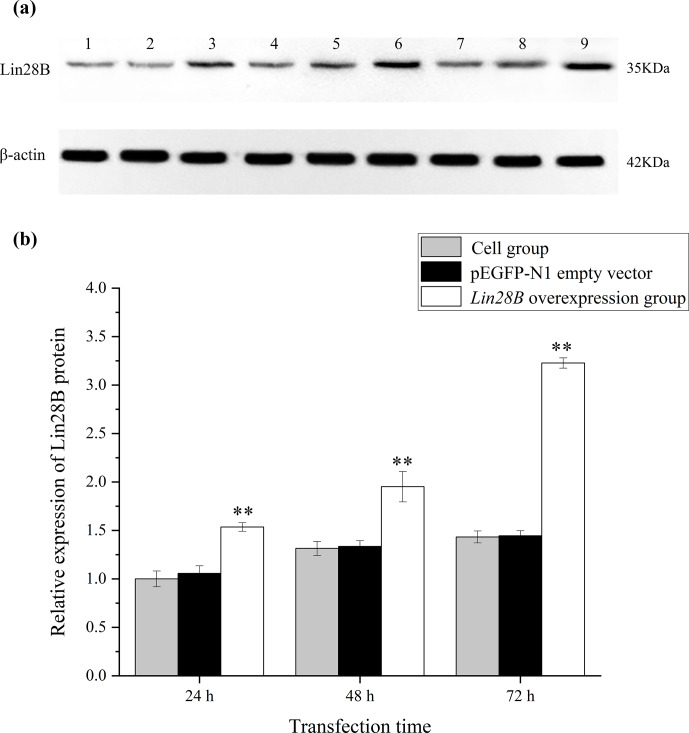
Changes in Lin28B protein expression at 24, 48, and 72 h after transfection. ${}^{**}$ P < 0.01. **(a)** Western blot strip diagram; **(b)** relative expression of Lin28B protein. Notes: (1) 24 h cell group; (2) 24 h empty-vector group; (3) 24 h *Lin28B* overexpression group; (4) 48 h cell group; (5) 48 h empty-vector group; (6) 48 h *Lin28B* overexpression group; (7) 72 h cell group; (8) 72 h empty-vector group; (9) 72 h *Lin28B* overexpression group.

## 3 Results

### 3.1 Identification of ovine GCs

Through cell immunofluorescence identification (Fig. 1), this test showed that FSHR was expressed in ovarian granulosa cells, as indicated by red immunofluorescence; DAPI staining appeared as blue (Hong et al., 2022; Wang et al., 2022). Upon merging these images, the blue fluorescence of DAPI and the red fluorescence of FSHR completely overlapped, indicating that the cells are of high purity, meeting the requirements of subsequent experiments.

**Figure 5:**
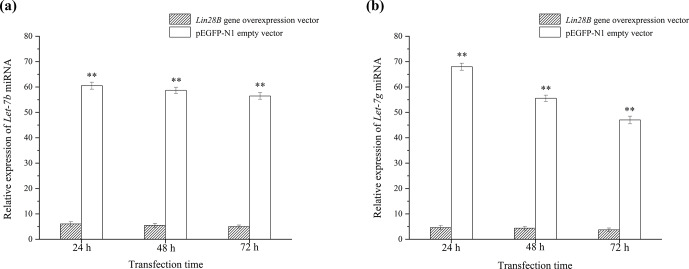
Effect of *Lin28B* overexpression on the expression of let-7b and let-7g. ${}^{**}$ P < 0.01. **(a)** Expression of *let-7b* after transfection with *Lin28B*; **(b)** expression of *let-7g* after transfection with *Lin28B*.

**Figure 6:**
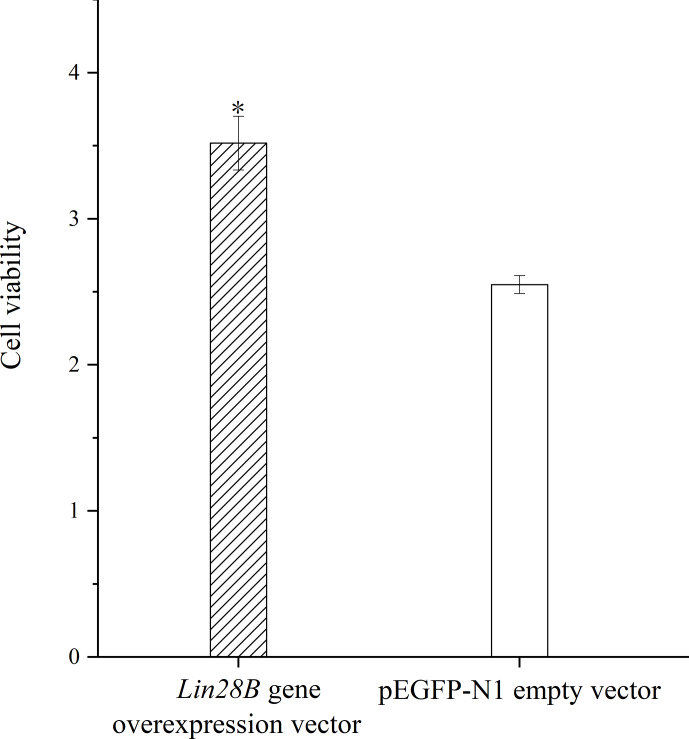
Effect of *Lin28B* overexpression on cell proliferation. ${}^{*}$ P < 0.05.

**Figure 7:**
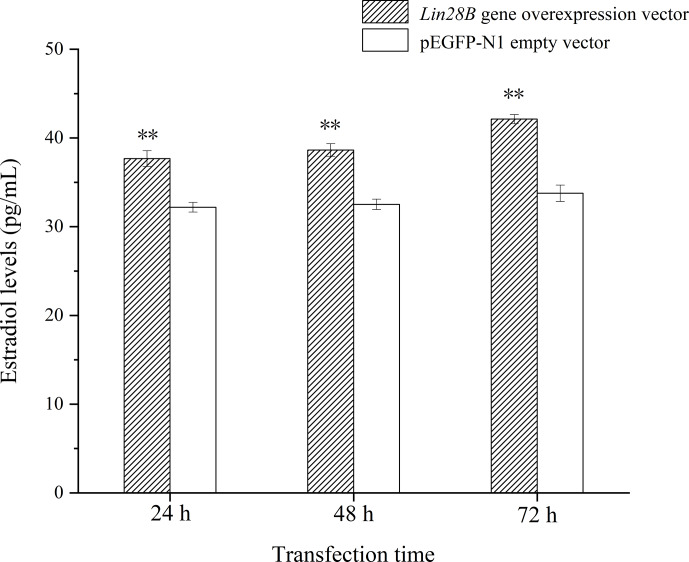
Effect of *Lin28B* overexpression on estradiol. ${}^{**}$ P < 0.01.

### 3.2 Expression of *Lin28B* in the granulosa cells of the ovaries

After transferring the *Lin28B* vector into ovarian granulosa cells, the fluorescence brightness of the test and control groups was examined at 24 h (Fig. 2), and *Lin28B* mRNA and protein expression levels were measured at 24, 48, and 72 h. After transfection, the mRNA (Fig. 3) and protein expression (Fig. 4) levels of the *Lin28B* gene in the overexpression group were significantly higher than those in the empty-vector group, indicating that the transfection was successful.

### 3.3 Effect of *Lin28B* overexpression on *let-7b* and *let-7g*

After cell transfection, the relative expression levels of *let-7b* and *let-7g* miRNA were measured using qRT-PCR (Fig. 5). The mRNA expression of the transfected *Lin28B* group was significantly higher than that of the empty-vector group ( P<0.01 ), indicating that *Lin28B* was successfully overexpressed in the ovarian granulosa cells. After the cells were transfected, the expression levels of *let-7b* and *let-7g* in the empty-vector group were significantly higher than those in the overexpression group ( P<0.01 ), which indicated that *Lin28B* overexpression significantly inhibited the expression of these miRNAs.

### 3.4 Effects of *Lin28B* overexpression on cell proliferation

Granulosa cell proliferation was measured with and without *Lin28B* overexpression. The overexpression of *Lin28B* significantly increased ovarian granulosa cell proliferation compared with the empty-vector group ( P<0.05 ; Fig. 6).

### 3.5 Effects of *Lin28B* overexpression on estradiol

Following *Lin28B* overexpression, estradiol secretion by granulosa cells was measured using ELISA. The cell culture medium from cells overexpressing *Lin28B* for 24, 48, and 72 h was collected. The detection of estradiol is shown in Fig. 7. The concentrations at 24, 48, and 72 h in this group were significantly higher than those in the empty-vector group ( P<0.05 ). *Lin28B* overexpression stably increased estradiol secretion at 24, 48, and 72 h. Estradiol levels in these periods were relatively stable, and the difference was not significant ( P>0.05 ).

## 4 Discussion

Puberty is the period when the reproductive ability of animals begins, and the length of puberty is related to the reproductive ability of animals (Xing et al., 2019b). Animals that reach puberty early have higher reproductive capacity and more offspring. Previous studies correlate puberty and *Lin28B* mRNA expression in livestock (Cao et al., 2020). To the best of our knowledge, this is the first study to investigate the role of *Lin28B* in sheep GCs. In the present study, an overexpression vector for *Lin28B* was constructed and transfected into GCs. The transfection was successful, as indicated by fluorescence imaging and relative mRNA expression. Following *Lin28B* overexpression, the *let-7b* and *let-7g* levels were found to be significantly lower than those of the control group. *Let-7b* and *let-7g* expression further decreased with increases in *Lin28B* expression. This result is consistent with *Lin28B* mRNA and *let-7b* miRNA expression in the hypothalamus of rats and female rhesus monkeys (Sangiao-Alvarellos et al., 2013).

Additionally, the present study showed that *Lin28B* overexpression promoted GC proliferation and E2 secretion. Other studies have shown similar results, even in cancer (Molenaar et al., 2012). According to related studies, the *Lin28B*–let-7 family axis regulates oncogenic cell function and the differentiation, growth, and metabolism of embryonic stem cells (Murray et al., 2013; Shyh-Chang and Daley, 2013). Aberrant expression of this axis is frequently observed in severe cancers, such as cervical, bladder, and lung cancers (Deng et al., 2017; Guo et al., 2017; Qi et al., 2018; Wu et al., 2019), and it normally regulates proliferative and metastatic functions. Furthermore, high *Lin28B* expression can promote the proliferation of human embryonic stem cells and accelerate their reprogramming process through faster cell division (Hanna et al., 2009; J. Yu et al., 2007). Let-7 miRNAs maintain differentiation patterns and normal development. Let-7 miRNAs and estrogen receptor (ER) α expressions are correlated (Sun et al., 2013). Estrogen secretion is suppressed by let-7 upregulation and decreases in ER α expression (Sun et al., 2016). The present study showed that *Lin28B* overexpression decreased *let-7b* and *let-7g* levels, promoted GC proliferation, and increased E2 secretion. Considering previous studies and our own, the *Lin28B*–let-7 family axis can be speculated to regulate GC proliferation and E2 secretion. However, the mechanism underlying this regulation should be investigated further.

## 5 Conclusions

In the present study, a vector overexpressing *Lin28B* was transfected into ovarian granulosa cells, whereby *let-7b* and *let-7g* miRNA levels decreased significantly. Concurrently, *Lin28B* overexpression can promote proliferation and estrogen secretion in sheep GCs. These findings can be used for further elucidating the regulatory mechanism of *Lin28B* in initiating sheep puberty.

## Data Availability

No data sets were used in this article.
